# Human bite as a weapon of assault

**DOI:** 10.4314/ahs.v18i1.12

**Published:** 2018-03

**Authors:** Samuel Ohayi Robsam, Emeka Udeh Ihechi, William Odesanmi Olufemi

**Affiliations:** 1 Histopathology ESUT College of Medicine Park lane, Enugu, Enugu 10003 Nigeria; 2 University of Nigeria, Enugu campus, Surgery; 3 Ekiti State University, Anatomic Pathology Ado Ekiti, Nigeria

**Keywords:** Human bite, teeth, weapon, assault, victim, biter

## Abstract

**Background:**

Human bite is a growing public health concern. It may be seen in both victims and aggressors following assault. Effects of human bite are both social and medical.

**Objectives:**

This study aims to determine the prevalence and characteristics of assault-related human bite injuries in Enugu, Nigeria.

**Methods:**

In this prospective study, an objective structured questionnaire was administered to assault victims attending the Forensic unit of ESUT Teaching Hospital between November 2013 and October 2014. Obtained data was analyzed.

**Results:**

Out of 219 patients presenting with clinical injuries, 29 (13.2%) sustained human bite wounds. Average age was 32.2±11.30 years and 34.3±12.4 years for victims and biters respectively. Females were more involved than males. Severe injuries resulted more in bites involving females than males. Contusion (47.6%) and laceration (31.0%) were the commonest. Upper limbs were mostly affected (44.7%) followed by the face (29.0%). Romance-related injuries affected breasts and thighs. Most incidents (62.1%) occurred within home/living quarters. Most biters were known to their victims.

**Conclusion:**

Human bite is a common outcome of assault and so should be anticipated in cases of assault. Patterns of location of bites seem related to nature of crime.

## Introduction

Human bite use as a weapon in assault is a growing public health concern. Effects of a human bite on a victim are both social e.g. cosmesis and medical e.g. infection. Human bite is found in violent crimes including child abuse, homicides, sexual assault and also in attempted suicide^1^. It may be found in the victim but also in an assailant as a defence wound from a victim^2^. It may occur in assaults, during sports events and may sometimes be intentionally self-inflicted to falsely frame someone^3^.

The biting teeth are usually the six front teeth namely the central incisors, lateral incisors and the cuspids^3^,^4^. Typically, a human bite injury is elliptical or circular in shape and records the specific characteristics of the inflicting teeth called tooth class characteristics which are features, characteristics, or patterns that distinguish a bite mark from other patterned injuries^5^. Also, it may be composed of two U shaped arches separated at their bases by an open space^3^. The diameter of the injury ranges from 25–40 mm^6^. When a bite injury is from only one of the two arches the pattern left usually provides very limited information to the investigator^3^.

Human bite mark analysis includes identifying a patterned injury as a human bite mark and secondly, pattern analysis of the bite mark to identify individual characteristics of the biting teeth which can provide information about the perpetrator^3^. Sample of perpetrator dentition is obtained from test bites where the suspect is made usually by a court order to bite inanimate objects and food stuff while saliva left on the bite site on the victim is swabbed for analysis for DNA^3^,^6^.

Bite marks can be classified into non-criminal (such as “love bites”) or criminal depending on the events that led to it. The criminal type can further be classified into offensive (upon victim by assailant) and defensive (upon assailant by victim)^7^,^8^. Human bite injuries may result from one of two mechanisms namely, the closed fist injury or “fight bite” (when the fist strikes a tooth or teeth with sufficient force to breach the integrity of the skin) or the occlusive bite injury (when the teeth close over with sufficient force as to breach and even avulse the tissue)^9^.

Any part of the human body may receive human bite injuries with circumstances of the injury, sex and age of the victim being factors that may affect the location of bites^10^. Human bites following sexual assault occur mostly on the face, lips, breasts, shoulder, neck, thighs, genitals and testicles; those following physical assault may be seen on any part of the body while defence wounds affect mostly the distal portions of the upper limb^3^,^10^–^12^. The circumstances that lead to human bite vary with age of victims with, for instance, those occurring in infants being usually punitive and often in response to crying and soiling while in childhood and older age, it usually follows assault^11^.

People with high risk for human bite include those confined to institutions like prisons and psychiatric hospitals, staff of such institutions, law enforcement agents^12^,^13^ and people who patronise “drinking joints” i.e. pubs^9^.

There are few works on human bite injuries in our environment and to the best of these authors' knowledge, there is none specifically focused on assault-related human bite injuries. This study therefore aims to highlight the characteristics of assault-related human bite injuries and thereby call the attention of concerned professionals and policy makers to this growing public health problem.

## Patients and methods

This was a prospective study. Pre-tested interviewer-administered objective structured questionnaires were used to collect data from assault victims attending the Forensic unit of Enugu State University Teaching Hospital, (ESUTH), Enugu between November 1, 2013 and October 31, 2014. Participants in the study were those who answered the interviewer's questions or filled out the questionnaire. Twenty nine (29) subjects completed the study out of which 10 were males and 19 were females.

**Setting:** The study was carried out in the Forensic unit Enugu State University Teaching Hospital, (ESUTH), Enugu. The unit which works in collaboration with appropriate hospital units and departments is dedicated to the forensic management of all cases of assault in the city of Enugu and its environs. The hospital itself is situated in the centre of the city. All the patients came to the unit upon referral by the police through a police medical report form D44. Data collected included victim and biter demographics, nature of bite injuries and circumstances surrounding the assault. Photographs of the bite marks were taken.

Ethical approval (ESUTHP/C-MAC/RA/012/Vol.07/02) was obtained from the hospital's ethics committee. Informed consent was obtained from patients while for those younger than 18 years, assent was obtained from them and informed consent from parents/guardians. Data obtained was entered into a Microsoft Excel spreadsheet and analysed. Frequency tables and bar chart were generated for selected variables. GraphPad Prism 5 software (GraphPad Software Inc., CA, USA was deployed for determining tests of statistical significance; for categorical variables and continuous variables Fisher's exact test or T test were used respectively. P value of <0.05 was considered as statistically significant.

## Results

A total of 1,877 persons whose demographic characteristics appear in Table 1 was seen in the Forensic unit during the study period. Two hundred and nineteen (11.7%) of them had clinical injuries and 29 (13.2%) of those with clinical injuries sustained human bite wounds. The bite injuries were inflicted in 29 incidents and by 31 biters.

**Table 1:** Demographic characteristics of all patients attending the Forensic unit

Age (years)	Male (%)	Female (%)	Total (%)
≥ 10	21 (1.1)	34 (1.8)	55 (2.9)
11 – 20	91 (4.8)	78 (4.2)	169 (9.0)
21 – 30	527 (28.1)	407 (21.7)	934 (49.8)
31 – 40	212 (11.3)	186 (9.9)	398 (21.2)
41 – 50	81 (4.3)	66(3.5)	147(7.8)
51 – 60	53 (2.8)	49 (2.6)	102 (5.4)
> 60	31 (1.7)	34(1.8)	65 (3.5)
Unknown	5 (0.3)	2 (0.1)	7 (0.4)

Total	1021 (54.4)	856(45.6)	1877 (100%)

Table 2 shows age and sex characteristics of victims and biters. For victims, age range was 15 years to 67 years and peak age was 21 years to 30 years (51.7%; n=15). The median age was 28 years while average age was 32.2±11.30 years. The average age for males and females was 32.1±12.74 years and 32.32±13.78 years respectively (p = 0.97). Of the biters, the age of three was unknown; the age range was 16 years to 60 years, peak age was 21 years to 40 years (68.5%; n=24) and median age was 30 years. While overall average was 35.54 years, average age for males and females was 39.14±11.58 years and 31.94±8.4 years respectively (p = 0.97).

**Table 2:** Distribution and analysis of age and sex of victims and biters

**Distribution of Victims and Biters by age and sex**						
	Victim			Biter		

Age (years)	Male	Female	Total	Male	Female	Total
	n (%)	n (%)	n (%)	n (%)	n (%)	n (%)
11 – 20	1 (3.45)	2 (6.9)	3 (10.3)	1 (3.2)	1 (3.2)	2 (6.5)
21 – 30	6 (20.7)	9 (31.0)	15 (51.7)	2 (6.5)	12(38.7)	14(45.2)
31 – 40	2 (6.9)	4 (13.8)	6 (20.7)	3 (9.7)	4 (12.9)	7 (22.6)
41 – 50	-	2 (6.9)	2 (6.9)	1 (3.2)	3 (9.7)	4 (12.9)
51 – 60	-	1 (3.45)	1 (3.5)	1 (3.2)	-	1 (3.2)
> 60	1 (3.45)	1 (3.45)	2 (6.9)	-	-	-
Unknown	-	-	-	1 (3.2)	2 (6.5)	3 (9.7)

Total	10(34.5)	19(65.5)	29 (100)	9 (29.0)	22(71.0)	31(100)

**Averages and tests of statistical significance**						
Sex	Victim		Biter		T test of means	Mean value of number of biter per victim
	Observed no. of biters	Mean age of biters	Observed no. of victims	Mean age of victims		
Male	10	32.1±12.74	9	39.14± 11.58	P=0.26	1.22±0.44
Female	19	32.31±13.78	22	31.94±8.4	P=0.92	1.5±0.92
T test of		P=0.97		P=0.97		
means						P=0.4

The male to female ratio was 1:1.9 and 1:2.4 for victims and biters respectively. Nineteen (19; 86.4%) female biters bit fellow females while 7 male biters (77.8%) bit fellow males. There was strong positive correlation (r = 0.78) between the gender of victims and biters (p = 0.001).

Table 3 shows the occupations of victims and biters. Female victims were mostly housewives, 5 (17.4%) while males were mostly artisans, 5 (17.4%). Most female biters (6:19.4%) were housewives.

**Table 3:** Occupation of victims and biters

Occupation	Victims			Biters		
	Male	Female	Total	Male	Female	Total
	n (%)	n (%)	n (%)	n (%)	n (%)	n (%)
Petty trader	1 (3.45)	4 (13.8)	5 (17.2)	-	5 (16.1)	5 (16.1)
Housewife	Not applicable	5 (17.2)	5 (17.2)	-	6 (19.4)	6 (19.4)
Civil servant	2 (6.9)	3 (10.35)	5 (17.2)	2 (6.45)	4 (12.9)	6 (19.35)
Professional	1 (3.45)	2 (6.9)	3(10.35)	1 (3.2)	-	1 (3.2)
Student	1(3.45)	2 (6.9)	3 (10.35)	2 (6.45)	1 (3.2)	3 (9.65)
Artisan	5 (17.2)	3 (10.35)	8 (27.6)	3 (9.7)	4 (12.9)	7 (22.6)
Unknown	-	-	-	1 (3.2)	2 (6.5)	3 (9.7)

Total	10(34.45)	19 (65.5)	29 (100)	9 (29.0)	22 (71.0)	31 (100)

**Fig. 1:**
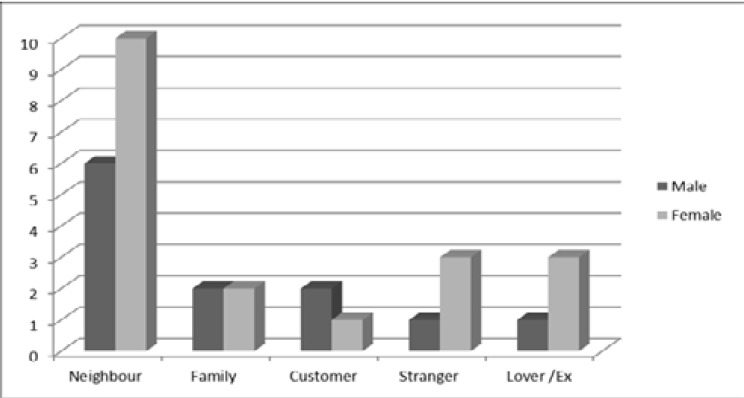
Relationship of biter to victim by sex

Figure 1 shows relationship of biters with their victims by sex. Twenty seven (87.1%) of the biters were well known to their victims while 4 (12.9%) were unknown to their victims before the assault. Of the known, 16 (51.6%) were neighbours (lived in the same tenement building) while 4 (12.9%) each were intimate partners and family members respectively.

The bites in our study occurred in 29 different incidents of assault from different circumstances (Table 4). Eighteen incidents (62.1%) occurred at the living quarters following conflicts between neighbours or people of the same household. Females were more victims and biters in home incidents. All 4 victims (13.8%) of romance-related bite injuries were females.

**Table 4:** Circumstance of human bite

Circumstance	Male (%)	Female (%)	Total (%)
Yard (living place)-related	4 (13.8)	10 (34.5)	14 (48.3)
Domestic dispute	2 (6.9)	2(6.9)	4 (13.8)
Business/Work related	2 (6.9)	2 (6.9)	4 (13.8)
Romance-related	-	4 (13.8)	4 (13.8)
Street/Drinking joint	1 (3.45)	1 (3.4)	2 (6.9)
Land dispute	1 (3.45)	-	1 (3.4)

Total	10 (34.5)	19 (65.5)	29 (100)

Table 5 shows injury type per victim and biter while table 6 shows anatomic distribution and frequency of injury in victims. A total of 42 bite wounds were seen giving an overall average of 1.5 bite wounds per victim. Females had more multiple bites with an average of 1.63±0.68 per person to 1.1±0.31 person for males (p=0.017).

**Table 5:** Injury type/severity in victims & biters (n=42)

	Victims				Biters			

Injury type	Male	Female	Total (%)	P	Male	Female	Total (%)	P
	(%)	(%)		value				value
Laceration	5(11.9)	8(19.1)	13 (31)	0.7	4(9.5)	9(21.4)	13 (31)	0.12
Amputation	1(2.4)	1(2.4)	2 (4.8)	1.0	1(2.4)	1(2.4)	2 (4.8)	1.0
Abrasion	1(2.4)	3(7.1)	4 (9.5)	0.48	2(4.8)	2(4.8)	4 (9.5)	1.0
Contusion	3(7.1)	17(40.5)	20 (47.6)	0.039	2(4.8)	18(42.8)	20 (47.6)	0.001
Avulsion	1(2.4)	2 (4.8)	3 (7.1)	1.0	2(4.8)	1(2.4)	3 (7.1)	1.0

Complex(>1 injury type)	1	6	7	0.08	2	5	7	0.56

**Fig. 2:**
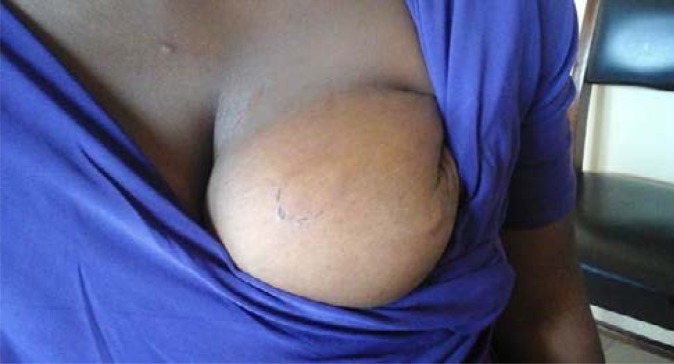
Contusion of the breast

**Fig. 3:**
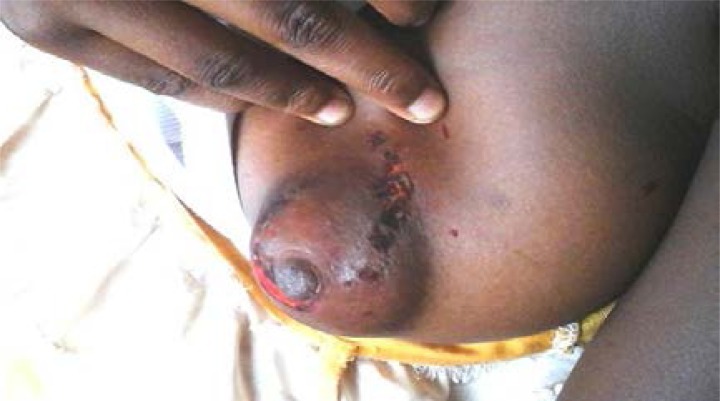
Abrasion and Laceration of the breast

**Fig. 4:**
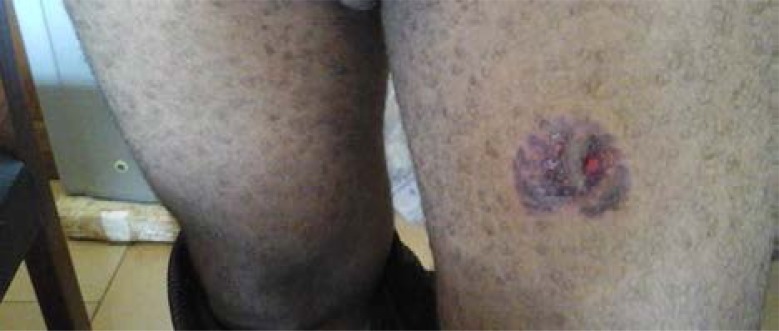
Abrasion of the thigh

Five injury types were identified with contusion (47.6%) as commonest (Fig. 2 Contusion of the breast). Others were laceration (31.0%) and abrasion (9.5%) shown in Fig. 3 (Abrasion and Laceration of the breast) and Fig. 4 Abrasion of the thigh.

**Fig. 5:**
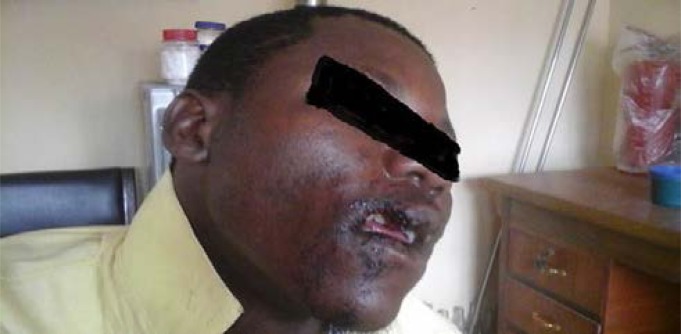
Upper lip amputation

**Fig. 6:**
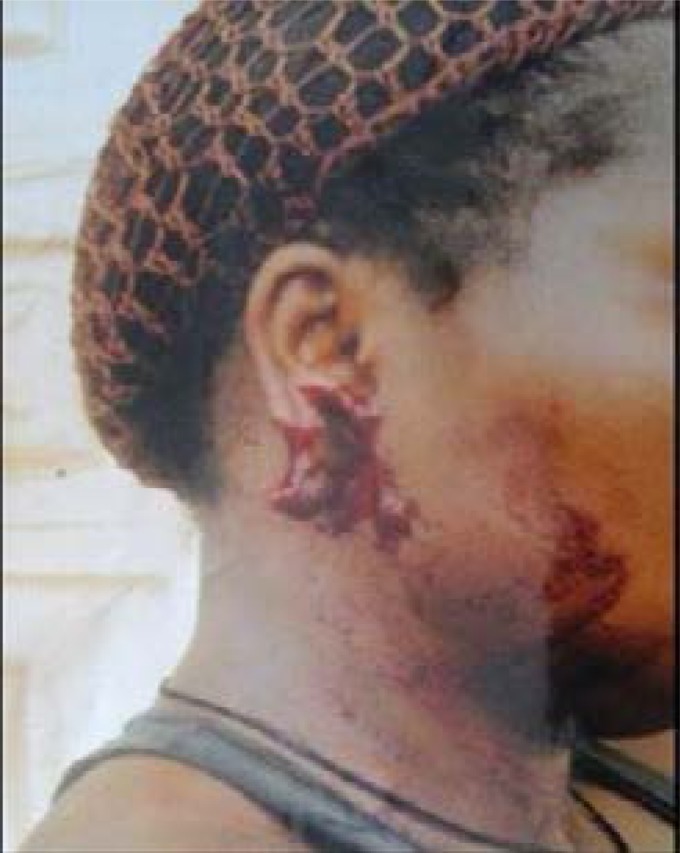
Right earlobe amputation

The rest were avulsion (7.1%) and amputation (4.8%) Fig. 5 (Upper lip amputation) and Fig. 6 (Right ear lobe amputation). Severe injuries namely laceration, avulsion and amputation occurred more in incidents involving females either as victims or as biters. The upper limbs (44.7%) were mostly affected followed by the face (29.0%). The left breast was involved twice; the right thigh twice and the scrotum once and all cases resulted from romance-related disputes. More females than males (10:1; p=0.029) were bitten on the face and at multiple sites (5:1; p=0.08). Males were bitten more on the hand than elsewhere.

Only 2 patients (6.9%) planned to pursue a legal action against their assailants. The rest were solely or mostly interested in recouping money they expended on treatment and servicing the police. The peak presenting time was 49 – 72 hours after the incident with most victims (n=24; 82.8%) presenting later than 12 hours post-incident. None of our patients reported self-inflicted or defence injuries.

## Discussion

Human beings have wittingly or unwittingly discovered the teeth as a veritable weapon for attack or defence in times of various emotions. This study shows a 13.2% incidence of usage of the teeth as assault weapon. Similar high incidences of human bite has also been reported from Tanzania^13^, the US and UK^14^,^15^ and Papua New Guinea^16^. The average age as well as the peak age range of victims in this study was similar to those reported in other studies^13^,^15^,^17^–^20^. Contrary to report by Freeman et al^10^, in our study the average ages of male and female victims were the same. Most victims and biters in our study were in active periods of life, a time they are more likely to be exposed to circumstances that engender assault. This is similar to reports from other studies^13^,^21^,^24^.

Our finding of more female victims and biters than males is not statistically significant (p=0.25 and 0.39 respectively) but agrees with a report from Zimbabwe^22^ and has some similarity with other studies^10^,^19^,^20^. It is however in disagreement with other studies which reported more male involvement^16^,^18^ or equal sex incidence in victims and biters^23^. Our finding that females tend to bite females more and males tend to bite males more (r=0.78) is statistically significant, p=0.001. The involvement of females more than males as biters may buttress the strong perception in our environment that women are physically weaker than men. Our study shows that most bites affect the arm followed by the face similar to the report by Freeman et al^12^ in which the arm is the most commonly affected followed by the leg, breast and face. However, other studies reported the face as the commonest site^9^,^14^,^24^.

The lip (27.3%) and ear (27.3%) are the most commonly affected parts of the face in our study in comparison with results from previous studies in Nigeria and Kenya^18^,^23^. We report a statistically significant (p=0.029) finding that females are more likely than males to be bitten in the face. Unlike other studies^13^,^23^, our study showed a high rate of bite injury involving the genital and peri-genital areas. Most of the facial bites like those to the breasts, scrotum and thighs resulted from romance-related conflicts. Multiple bites/sites of injury were more likely to occur in females than males (P=0.029). Contusion is commonest in our study similar to other studies^13^,^14^ and is more likely to be seen in female bites than males (p=0.039). In our study, females suffer high numbers of bites than males (p=0.017). Similar to reports from other workers^10^,^25^, this study shows that patterns of location, number and severity of injuries correlate with circumstance of crime and age and sex of victim. It would appear that biters target the face and the breast of victims, both central to romantic attractions, possibly to degrade the worth of the opponent in such affairs. Also, the upper limb received frequent human bite wounds probably because they are inevitably employed in cases of interpersonal violence being the primary organ of grasping.

Most of our patients were known to their biters. This agrees with other studies^17^,^18^. Similarly, conflicts around the home/living quarters formed the largest source of human bite injuries. Our patients were from various social strata similar to the finding by Olaitan^20^. Though Eardley et al^24^ had established a clear link between weekend drinking and human bite injuries, none of our patients gave a history of alcohol use prior to the bite. It was difficult to affirm or disprove this since most patients presented late. As in other studies^17^,^20^,^24^, the reasons for assault-related human bite injuries varied widely in our study and include jealousy, household or workplace disputes and matrimonial disputes. These factors appear amplified by social tension and competition that follow over-crowding and harsh economic conditions.

Although all our patients had reported their injuries to the police, like other Nigerian studies^18^,^20^, a very small proportion of our patients planned to seek legal redress. Most were more interested in recouping money they expended or lost for various reasons following the assault. It seems that sociocultural system affects the attitude of assault victims towards seeking legal redress. The reasons given by our patients for their apathy towards legal include intervention by religious or sociocultural/community leaders, misgivings about the police, perceived slowness of the legal process and ignorance of the law. An overwhelming 82.7% (n=24) of our patients presented more than 12 hours after injury with the peak presentation time being 49 – 72 hours post-injury. This is similar with other studies^9^,^24^. Delays are mostly attributed to police attitude, seeking help from patent medicine dealers and mediatory attempts by third parties among others.

Prosecution of assault-associated human bite offers some unique challenges in a resource-limited setting like ours. The only additional tool (other than medical history and examination) the forensic physician may have is photograph(s) of the bite injury. Facilities for DNA recovery and making of casts for the bite injury as well as the suspect's teeth are lacking. Also, there are no forensic odontologists. Thus the evidential value of human bite in our environment is highly limited. Pretty and Sweet^26^ may have had our kind of situation in mind when they concluded following a review of past cases of human bite that in addition to its forensic values, bite mark analysis may also be the enemy of natural justice.

## Conclusion

Human bite is readily used as a weapon of assault in our environment. As such, people presenting with assault should be properly evaluated for human bite and vice versa. Also, the location of human bite may point to the circumstances leading to the injury. There is need to educate the society on the need to seek legal redress following assault-related human bite and other injuries.

## Figures and Tables

**Table 6:** Anatomic distribution and frequency of injury in victims

**Anatomic distribution in victims (n=29)**				

Site	Male (%)	Female (%)	Total (%)	P value (Fisher's exact test)
Face	1(20.7)	10 (34.4)	11 (37.9)	P=0.029
Upper limb	6 (20.7)	10 (34.4)	16 (55.2)	P=0.70
Breast	-	2 (6.9)	2 (6.9)	P=0.33
Trunk	3 (10.3)	2 (6.9)	5 (17.2)	P=0.76
Genitalia	1 (3.4)	-	1 (3.4)	P=1
Thigh	-	2 (6.9)	2 (6.9)	P=0.33
Multiple sites	1 (3.4)	5 (17.2)	6 (20.7)	P=0.029

**Number of injury per victim by sex (n=42)**					

		No. of injuries		No. of bites/	Mean value of
Sex	1	2 – 3	> 3	No. of victims	bites per victim
Male	9	1	-	11/10	1.1±0.31
Female	13	4	2	31/19	1.63±0.68

T test for mean					P=0.017
